# A serum microRNA signature associated with complete remission and progression after autologous stem-cell transplantation in patients with multiple myeloma

**DOI:** 10.18632/oncotarget.2761

**Published:** 2015-01-22

**Authors:** Alfons Navarro, Tania Díaz, Natalia Tovar, Fabiola Pedrosa, Rut Tejero, María Teresa Cibeira, Laura Magnano, Laura Rosiñol, Mariano Monzó, Joan Bladé, Carlos Fernández de Larrea

**Affiliations:** ^1^ Molecular Oncology and Embryology Laboratory, Human Anatomy Unit, School of Medicine, University of Barcelona, 08036 Barcelona, Spain; ^2^ Amyloidosis and Myeloma Unit, Department of Hematology, Hospital Clínic de Barcelona, Institut d'Investigacions Biomèdiques August Pi I Sunyer (IDIBAPS), University of Barcelona, 08036 Barcelona, Spain

**Keywords:** miRNA, serum, myeloma, autologous transplantation, complete remission

## Abstract

We have examined serum microRNA expression in multiple myeloma (MM) patients at diagnosis and at complete response (CR) after autologous stem-cell transplantation (ASCT), in patients with stable monoclonal gammopathy of undetermined significance, and in healthy controls. MicroRNAs were first profiled using TaqMan Human MicroRNA Arrays. Differentially expressed microRNAs were then validated by individual TaqMan MicroRNA assays and correlated with CR and progression-free survival (PFS) after ASCT. Supervised analysis identified a differentially expressed 14-microRNA signature. The differential expression of miR-16 (*P* = 0.028), miR-17 (*P* = 0.016), miR-19b (*P* = 0.009), miR-20a (*P* = 0.017) and miR-660 (*P* = 0.048) at diagnosis and CR was then confirmed by individual assays. In addition, high levels of miR-25 were related to the presence of oligoclonal bands (*P* = 0.002). Longer PFS after ASCT was observed in patients with high levels of miR-19b (6 vs. 1.8 years; *P* < 0.001) or miR-331 (8.6 vs. 2.9 years; *P* = 0.001). Low expression of both miR-19b and miR-331 in combination was a marker of shorter PFS (HR 5.3; *P* = 0.033). We have identified a serum microRNA signature with potential as a diagnostic and prognostic tool in MM.

## INTRODUCTION

Multiple myeloma (MM), a malignant disorder characterized by the neoplastic growth of bone marrow plasma cells, accounts for 15% of all hematologic cancers [1, 2]. MM usually progresses from an asymptomatic premalignant state of clonal plasma cell proliferation known as monoclonal gammopathy of undetermined significance (MGUS) [3]. Although the introduction of new drugs, such as thalidomide, bortezomib and lenalidomide, has resulted in a superior complete response (CR) rate and longer progression-free survival (PFS) [4], MM is still an incurable disease. At present, the monitoring of response and progression requires an invasive procedure for the examination of bone marrow, since tumor cells are localized in the marrow and do not usually circulate in blood [5]. However, extramedullary disease cannot be accurately evaluated with bone marrow studies [6], and moreover, there is a need for non-invasive techniques for both the initial diagnosis and the follow-up of patients with MM. To date, although serum-free light chain ratio [7] is a well-recognized biomarker for monitoring patients with MM and MGUS, only few other serum-based markers for MM have been incorporated into routine clinical practice.

MicroRNAs (miRNAs) are small endogenous non-coding RNAs that regulate gene expression, mainly by inhibiting translation of specific target mRNAs. miRNAs are involved in the regulation of several physiological and pathological processes, including tumorogenesis [8] and have emerged as promising prognostic biomarkers and as possible therapeutic targets [9]. Since it was first discovered that miRNAs can also be detected in serum or plasma [10, 11], research on circulating miRNAs has intensified and led to the development of a database of human circulating miRNAs [12]. In recent years, several studies have identified miRNA profiles in human myeloma cell lines and in primary patient samples, and the miRNA expression pattern has been associated with specific genetic abnormalities [13–16] and with patient survival [13, 17]. Interestingly, some miRNAs, such as the miR-17-92 cluster, are differentially expressed in multiple myeloma and MGUS [18]. The first promising biomarker detected in plasma from patients with MM was miR-92a, which was downregulated in MM but not in MGUS or smoldering MM [19]. Other studies have also identified several circulating miRNAs that show promise as potential prognostic or predictive markers [20–22]. Recently, miR-16 and miR-25 have been proposed as independent prognostic markers in newly diagnosed MM [23]. Taken together, these findings indicate that circulating serum miRNAs are an easily accessible, stable biomarker that could meet the need for a non-invasive method for monitoring patients with MM.

In order to further explore this potential role of circulating serum miRNAs, we have analyzed the expression of miRNAs in serum from three cohorts of individuals: paired samples from patients with MM at the time of diagnosis and of CR after autologous stem-cell transplantation (ASCT) (Table 1); samples from MGUS patients; and samples from healthy controls. In addition, in a subset of the patients with MM, we have compared miRNA expression levels at the time of CR and of relapse.

**Table 1:** Characteristics of the 33 patients with multiple myeloma included in the first part of the validation phase

Characteristic	
**Median age, yrs (range)**	56 (25–66)
**Gender (M/F)**	13/20
**Immunological subtype**	
IgG	39.4%
IgA	24.2%
Light chains	24.2%
Oligosecretory	3%
Others	9.2%
**Light chain subtype**	
kappa	57.6%
lambda	39.4%
International Staging System^*^	
I	51.6%
II	32.3%
III	16.1%
**Median bone marrow plasma cells**	44%
**Extramedullary involvement**	36.4%
**Hemoglobin <10 g/L**	21.2%
**Calcium ≥11.5 mg/dL**	12.1%
**Creatinine ≥2 mg/dl**	9.1%
**Lytic bone lesions**	66.7%
Induction treatment^**^ Polychemotherapy (M2/VBAD, VAD, CY/Dex) Thalidomide/dexamethasone Bortezomib/thalidomide/dexamethasone Other bortezomib/dexamethasone-based	32.3% 19.3% 12.9% 35.5%

* Available in 31 patients

** One patient underwent autologous SCT without previous cytoreductive treatment and one received only local radiotherapy

## RESULTS

### Screening phase: identification of miRNAs differentially expressed

**Figure 1:**
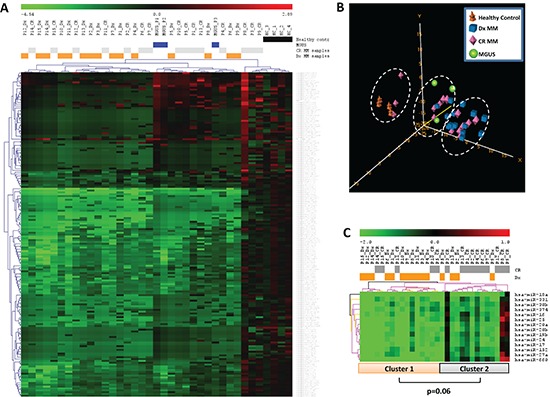
miRNA expression pattern in serum samples from patients with multiple myeloma (MM), patients with monoclonal gammopathy of undetermined significance (MGUS), and healthy controls **(A)** Unsupervised hierarchical cluster analysis and **(B)** principal component analysis identified three major clusters with different patterns of miRNA expression. **(C)** Hierarchical cluster analysis of 14 miRNAs that were underexpressed in the diagnosis (Dx) samples compared to the complete remission (CR) samples from MM patients identified two clusters.

The unsupervised hierarchical cluster analysis showed three major clusters with different patterns of miRNA expression (Figure 1A). The first cluster included all healthy control samples and three CR samples. The second cluster included three MGUS samples, eight CR samples and four diagnostic samples. Finally, the third cluster included 11 diagnostic samples and seven CR samples. A gradual decrease in overall miRNA expression levels was observed from the first to the third cluster (Figure 1A). PCA also distinguished between the three groups identified in the hierarchical cluster analysis (Figure 1B).

#### 14-miRNA signature in multiple myeloma

Supervised analysis by SAM and *t*-test of the paired samples (diagnostic vs. CR) of 15 patients with MM revealed 14 miRNAs that were underexpressed in the diagnostic samples compared to the CR samples (Table 2). The hierarchical cluster analysis of these 14 miRNAs identified two clusters. The first cluster included 74% of the diagnostic samples, while the second cluster included 67% of the CR samples (*P* = 0.06) (Figure 1C).

**Table 2:** Significance analysis of microarrays (SAM) and Student's *t*-test identified 14 miRNAs differentially expressed in serum from myeloma patients at diagnosis and the time of complete response

miRNA	Adjusted *P*-value	Fold Change
miR-16	2.244784E-4	0.005676097
miR-17	5.8513036E-5	0.0051784036
miR-18a	0.0074316533	0.03986069
miR-19b	2.913102E-4	0.0051561906
miR-20a	8.366445E-4	0.008710946
miR-20b	4.931529E-4	0.0067144665
miR-24	1.8698742E-4	0.0066193547
miR-25	6.002177E-4	0.0066399085
miR-27a	0.0011549154	0.010221002
miR-30b	5.4020213E-4	0.0068296986
miR-152	0.006841648	0.039063603
miR-331	9.056236E-4	0.008436599
miR-374	3.6963476E-5	0.0065425355
miR-660	5.7780603E-4	0.006818111

### Validation phase I: confirmation of differential expression of miRNAs at diagnosis and at CR

#### miR-16, miR-17, miR-19b, miR-20a and miR-660 as markers of CR

**Figure 2:**
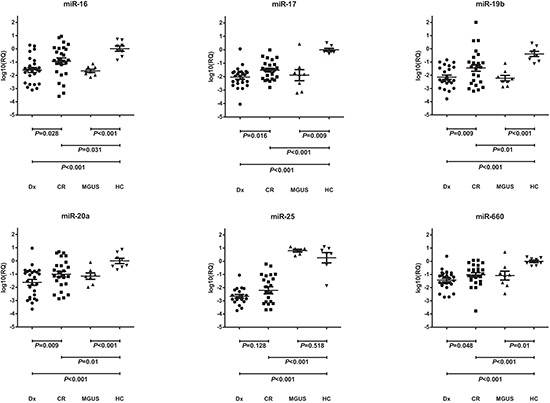
Differential serum levels of miR-16, miR-17, miR19b, miR-20a, miR-25 and miR-660 in patients with multiple myeloma (MM) at diagnosis (Dx) and at complete remission (CR), in patients with monoclonal gammopathy of undetermined significance (MGUS), and in healthy controls (HC)

The analysis of the 14 miRNAs identified in the screening phase confirmed the differential expression of five miRNAs between the diagnostic and CR samples of the patients with MM: miR-16 (*P* = 0.028), miR-17 (*P* = 0.016), miR-19b (*P* = 0.009), miR-20a (*P* = 0.017) and miR-660 (*P* = 0.048) (Figure 2). Patients in CR showed a partial recovery of the normal serum levels of these five miRNAs. Levels in samples from MGUS patients were similar to those in CR samples but lower than healthy control samples (Figure 2). The ANOVA test showed significant differences between control, MGUS, diagnostic and CR samples in the expression levels of miR-16 (*P* < 0.001), miR-17 (*P* < 0.001), miR-19b (*P* < 0.001), miR-20a (*P* = 0.002), miR-660 (*P* < 0.001) and miR-25 (*P* < 0.001), with the highest levels of expression observed in samples from healthy controls. Although miR-25 was underexpressed in patients with MM compared to MGUS patients and healthy controls, among patients with oligoclonal bands miR-25 expression was higher than in the other patients in CR without serum oligoclonal humoral response (*P* = 0.002). We also observed a trend towards lower miR-25 levels in diagnostic samples from patients with lytic bone lesions than in those without them (*P* = 0.07).

#### miR-19b and miR-133 as markers of PFS after CR

Twenty-eight of 33 patients with MM showed oligoclonal bands in serum and/or urine while in CR. At the time of analysis, 18 patients (54%) had relapsed after ASCT. Median PFS for all 33 patients was 5 years (95% CI, 2.2–73.8) and median overall survival was not reached (estimated survival at 5 years, 88.7%).

**Figure 3:**
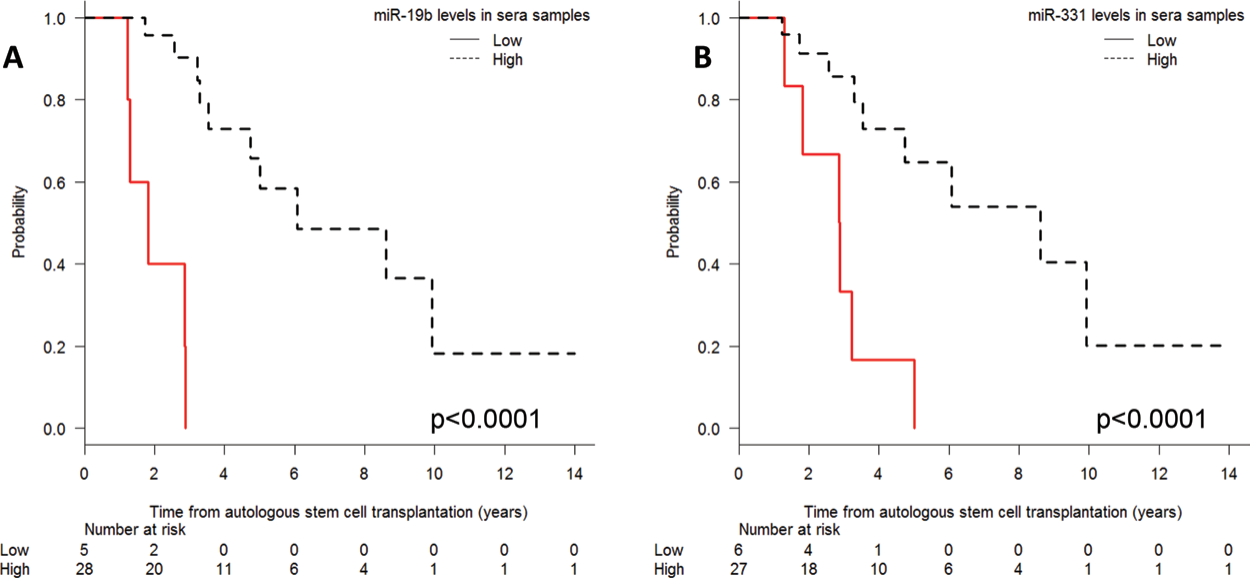
Progression-free survival after autologous stem-cell transplantation according to (A) miR-19b and (B) miR-331 expression levels in serum

**Figure 4:**
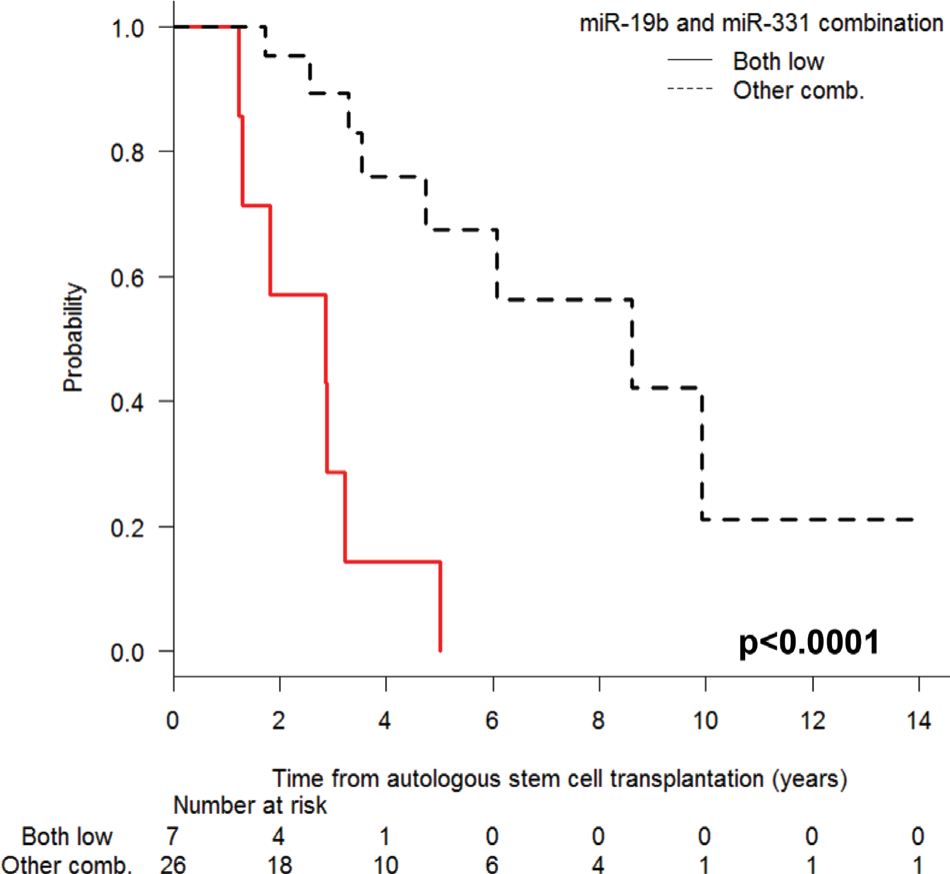
Progression-free survival after autologous stem-cell transplantation according to the expression levels of miR-19b and miR-331, comparing patients with low levels of both miRNAs and those with high expression of either miRNA

Shorter PFS was associated with low miR-19b levels (median 1.8 vs. 6 years; *P* < 0.001) and low miR-331 levels (median 2.9 vs. 8.6 years; *P* = 0.001) at the time of CR (Figure 3). Moreover, when we examined the combinatory effect of these two miRNAs, we found that PFS was shorter (*P* < 0.001) in patients with low levels of both miRNAs than in those with high levels of either one (Figure 4).

In the univariate analysis, only older age (>55 years), high creatinine levels (>2 mg/dL), and low miR-19b/miR-331 expression were associated with shorter PFS. The multivariate analysis identified the miR-19b/miR-331 combination (HR, 5.3; 95% CI, 1.1–24.7; *P* = 0.033) and creatinine levels (HR, 7.5; 95% CI, 1.9–29.7; *P* = 0.004) as prognostic markers of PFS.

### Validation phase II: comparison of serum miRNA levels at CR and at relapse

In the subset of 17 patients with paired serum samples at CR and at relapse, we examined the expression levels of the miRNAs that had been related to CR or PFS in the first part of the validation phase. Significantly lower levels of miR-19b were observed in samples obtained at relapse than in those obtained at CR (*P* = 0.04). A non-significant trend towards a difference in miR-331 expression was observed, and there were no significant differences between expression levels of miR-16, miR-17, miR-20a, miR-25 or miR-660.

## DISCUSSION

Biomarkers have a clear role in assessing response to treatment and prognosis in patients with malignant monoclonal gammopathies, as well as in therapeutic decision-making and early diagnosis in oligosymptomatic cases [24]. When these biomarkers can be examined in plasma or serum, they can potentially be used to evaluate both medullar and extramedullar disease at different time points in a non-invasive manner. For example, serum-free light chain assay is a commonly used method for monitoring patients [25], and recently, other more innovative techniques have been described, including next generation sequencing in peripheral blood [26], proteomics for sensitive measuring of M-protein [27], and circulating miRNAs [23]. Circulating miRNAs are particularly promising biomarkers, since they remain stable for long periods of time, are relatively easy to measure, and reflect the entire tumor mass, including plasma cells that are either absent or patchy in bone marrow [28].

In the present study, we have quantified circulating miRNAs in serial serum samples from patients with MM and in samples from MGUS patients and healthy controls and have identified a 14-miRNA signature which was underexpressed in patients with MM, while MGUS patients showed an intermediate expression level between patients with MM and healthy controls. Moreover, the expression of five of these miRNAs – miR-16, miR-17, miR-19b, miR-20a and miR-660 – increased at the time of CR, and two of the 14 miRNAs – miR-19b and miR-331 – were linked to PFS after ASCT. Patients with lower levels of miR-19b or miR-331 had shorter PFS than those with higher levels, and moreover, those with low levels of both miRNAs had shorter PFS than those with high levels of either miRNA. Importantly, the miR-19b/miR-331 combination retained its prognostic value in the multivariate analysis (HR, 5.3; *P* = 0.033).

The components of the 14-miRNA signature can be classified in five expression clusters: miR-16, miR-17-92 cluster and paralogs, miR-23/24/27a, miR-331 and miR-660. The origin and mechanism of action of these miRNAs are not clear; however, since all 14 miRNAs are downregulated in serum, we can speculate that their origin is not in the tumor cell. In fact, serum miRNA showed no correlation with intracellular levels in malignant bone marrow plasma cells in paired samples [23]. Several cells, including those in blood and in the bone marrow microenvironment, can release miRNAs or modify their expression in response to MM. It is well known that almost 80% of MM patients develop osteolytic lesions. MM cells stimulate bone resorption by enhancing osteoclastogenesis, while suppressing bone formation by inhibiting osteoblastic differentiation from bone marrow stromal cells, leading to extensive bone destruction with rapid bone loss [29]. Several miRNAs included in our 14-miRNA signature have previously been related to osteoblasts, leading us to speculate that their downregulation in patients with MM could be due to the reduction of osteoblasts associated with the disease. For example, miR-16 has previously been found elevated during osteoclast/osteoblast differentiation [30] and its ectopic expression in serum correlated with bone metastasis burden in breast cancer [31]. In addition, the miR-17-92 cluster and paralogs, which includes miR-25, have been related to the regulation of osteoblast proliferation and differentiation [32] and to the promotion of hematopoietic cell expansion by augmenting HIF-1a in osteoblasts [33]. Interestingly, a mouse model with deletion of miR-17-92 cluster showed reduced skeletal growth with delayed ossification likely due to impaired osteoblast proliferation and differentiation [34]. Along these lines, in the present study, there was a trend towards lower levels of miR-25 in patients who had bone lesions at diagnosis. Finally, miR-27, part of the miR-23/24/27a cluster, activates the Wnt signaling pathway, affecting the differentiation of mesenchymal stem cells into osteoblasts [35]. All these data suggest a potential role for the osteoblast/osteoclast axis in circulating miRNA levels that should be explored in prospective studies along with bone remodeling biomarkers. miRNAs can be contained within exosomes, and our knowledge of their role in communication between cells is growing. These vesicles of endocytic origin can be released by many types of cells, facilitating several biological processes [36]. In fact, bone marrow mesenchymal cells can deliver exosomes to MM cells, and exosomes from patients with MM have lower levels of the tumor suppressor miR-15 and higher levels of oncogenic proteins, cytokines, and adhesion molecules [37]. This highlights the importance of the bone marrow microenvironment in miRNA homeostasis in MM activity and remission.

In our patients with MM, several miRNAs were underexpressed in serum at diagnosis but increased at CR; moreover, miR-19b and miR-331 were associated with shorter PFS. Our findings are in line with several other studies relating miRNAs, including those in our 14-miRNA signature (Table 2) and others, to prognosis in MM and other hematological and solid tumors. For example, downregulation of circulating miR-16 and miR-25 were related to poor prognosis in multiple myeloma [23]. Interestingly, miR-16 has a tumor suppressor role in MM pathogenesis by targeting clonal plasma cells, reducing bone marrow neoangiogenesis and reducing interaction between the tumor cells and the bone marrow. miR-16 induced a 60% reduction in MM cells proliferation at 72 h after pre-miR-16 transfection [38]. Moreover, downregulation of miR-331-5p and miR-27a was associated with chemotherapy resistance and relapse in leukemia [39]. Downregulation of circulating miR-660 has been reported in plasma from lung cancer patients [40].

In another study, three miRNAs (miR-720, miR-1308 and miR-1246) showed a good specificity in distinguishing MM or MGUS from healthy individuals and also in differentiating MM from MGUS [21]. A microarray analysis found six miRNAs (miR-148a, miR-181a, miR-20a, miR-221, miR-625 and miR-99b) that were upregulated in patients with MM compared to healthy controls [20]; miR-221 and miR-99b were related to the karyotype of the disease, whereas miR-148a and miR20a were related to relapse-free survival [20]. Recently, Kubiczkova et al have identified a miRNA profile deregulated in MM and MGUS serum, where miR-744, miR-130a, let-7d and let-7e levels were decreased and miR-34a levels were increased in MM and MGUS when compared to healthy controls [22]. Additionally, miR-744 and let-7e levels were biomarkers of overall survival [22].

Our study differs in scope from previously reported series, particularly that by an Italian group in 54 patients [23]. Firstly, all of our 33 patients underwent ASCT, while the patients in the Italian study were older and thus ineligible for stem-cell transplantation. In addition, the main goal in the Italian study was to examine the prognostic impact of the serum expression of miR-16 and miR-25 at diagnosis, together with cytogenetics by FISH and ISS, which were already well established as prognostic factors, and to compare the expression of these miRNAs in 32 paired samples of serum and bone marrow plasma cells. In contrast, we have compared the serum miRNA profile in paired samples at diagnosis and at the time of CR in order to define the prognostic impact of serum miRNA for patients in CR. Since a long-lasting CR is crucial for survival of patients with MM [41, 42], serum biomarkers to predict the duration of CR are clearly relevant in this setting.

As with all studies, ours has several limitations. Since we selected patients with samples at the time of CR, our series was enriched with more sensitive patients and our findings should be explored in refractory and high-risk patients, particularly those with extramedullary disease. In addition, the retrospective nature of our study and the relatively small number of patients included make it difficult to draw definitive conclusions from our findings; further studies in our cooperative Spanish group and prospective validation in sequential samples are therefore warranted. However, despite these limitations, our results highlight the potential value of serum miRNAs as diagnostic and prognostic biomarkers in MM and pave the way for further studies of our 14-miRNA signature in larger, more varied patient cohorts.

## PATIENTS AND METHODS

### Study design and patients

The study consisted of an initial screening phase, followed by a two-part validation phase. The objective of the screening phase was to identify miRNAs differentially expressed in serum samples from patients with MM at the time of diagnosis, the same patients at the time of CR, MGUS patients, and healthy controls. In the first part of the validation phase, the miRNAs identified in the screening phase were analyzed individually to confirm their differential expression between patient samples at diagnosis and at CR. Finally, in a subset of patients, the second part of the validation phase compared miRNA expression at the time of CR and of relapse.

The screening phase included 15 patients with MM, four patients with stable MGUS (>5 years of follow-up), and five healthy controls. Paired serum samples were obtained from the patients with MM at diagnosis and at the time of confirmed CR after melphalan-based ASCT.

In the first part of the validation phase, a total of 33 patients with MM, eight MGUS patients and eight healthy controls were included. The main clinical characteristics of the patients with MM are summarized in Table 1. Median follow-up for alive patients was four years (range, 1–14). In these patients, cytogenetic abnormalities by FISH in bone marrow plasma cells were t(4;14) in four, t(11;14) in four, t(14;16) in one, and del17p in one patient. Nine patients had del13q by conventional karyotype. Information on time to relapse after ASCT, further treatment and follow-up was collected for all patients. The second part of the validation phase was performed on the subset of 17 patients with MM with available paired serum samples at CR and at the time of relapse after ASCT.

Response and relapse were defined according to European Blood and Marrow Transplantation criteria [43]. In short, CR required the disappearance of the original myeloma protein in serum and urine immunofixation (IFE) and less than 5% bone marrow plasma cells. In patients with CR, relapse was defined by recurrence of detectable-component on IFE, even if electrophoresis remained negative, excluding oligoclonal immune reconstitution.

Written informed consent was obtained from each participant in accordance with the Declaration of Helsinki and the Ethics Committee of Hospital Clínic of Barcelona provided institutional review board approval for this study.

### RNA extraction and miRNA quantification

RNA was extracted from frozen serum samples using miRNeasy Mini Kit (Qiagen). In the screening phase, 380 miRNAs and controls were profiled using TaqMan Human MicroRNA Arrays (Array A; Applied Biosystems) using pre-amplification as per the manufacturer's protocol. Expression levels were calculated by the 2^−ΔΔCt^ method. Real-time quantitative PCR reactions were performed on an ABI 7900 HT Sequence Detection System (Applied Biosystems). miR-483-5p was used as endogenous control, since this miRNA had the smallest standard deviation among samples and showed no statistical differences in its expression levels between patients and healthy controls. The median level of healthy controls was used as calibrator. All miRNAs that expressed <10% or with an unreliable quantification were excluded from further analysis, leaving a set of 177 miRNAs.

In the validation phases, miRNAs differentially expressed between diagnosis and CR or between CR and relapse were selected for validation by single Real Time TaqMan^®^ MicroRNA Assays (without pre-amplification) in an Applied Biosystems 7500 Sequence Detection System as previously described [44, 45].

### Statistical analysis

In the screening phase, data on miRNA expression were analyzed using BRB Array Tools version 3.5.0 software (Richard Simon & BRB-ArrayTools Development Team, http://linus.nci.nih.gov/BR-ArrayTools.html), TIGR Multiexperiment viewer version 4.0 software (The Institute for Genomic Research, and ArrayAssist software, Stratagene, http://www.tm4.org/mev), R software version 2.15.2 (The R Foundation for Statistical Computing c/o Institute for Statistics and Mathematics, Wirtschaftsuniversität Wien, http://www.-project.org/) and GraphPad software 5.0 (GraphPad Software Inc., San Diego, CA, USA, http://www.graphpad.com/). Hierarchical clustering (using Manhattan distance and average linkage) and principal component analysis (PCA) were used to classify samples. To identify miRNAs with significant differential expression between groups, two multivariate permutation tests were performed: significance analysis of microarrays (SAM) and Student's *t*-test based on multivariate permutation (with random variance model). Differences between miRNAs in the screening phase of the study were considered statistically significant if the *t*-test showed *P* < 0.001 or SAM showed a false discovery rate of <0.1% (Supplementary Table 1). A final miRNA signature comprised miRNAs that were identified by both methods.

In the validation phases, the expression levels of individual miRNAs were compared between groups using paired or unpaired *t*-test or ANOVA analysis for continuous variables. For patients with CR, PFS was defined as survival from the time of ASCT until relapse or death from any cause. Overall survival was calculated from the time of ASCT to last follow-up or death from any cause. Optimal cutoffs of miRNA expression were assessed by means of maximally selected log-rank statistics using the Maxstat package (R package). Survival probabilities were estimated using the Kaplan-Meier method and compared by means of the log-rank test. The Cox proportional hazards model was used to estimate the hazard ratios (HR) with their respective confidence intervals (CIs) after controlling for prognostic variables.

## SUPPLEMENTARY TABLE


