# Supplementary material for: A serum microRNA signature associated with complete remission and progression after autologous stem-cell transplantation in patients with multiple myeloma
Source: Oncotarget. 2015 Jan 22;6(3):1874–83. doi: 10.18632/oncotarget.2761 (PMC4359338; doi:10.18632/oncotarget.2761)
Supplement: Supplementary file 1 [file oncotarget-06-1874-s001.pdf]

SUPPLEMENTARY TABLE

Supplementary Table 1: miRNAs identified by SAM and *T*-test during the profiling phase. In Bold are the microRNAs that were identified by both methods

microRNA	Mean expression at Dx	SD at Dx	Mean expression at Remision	SD at Remision	Adjusted *p*-value	Fold change
**hsa-miR-374**	**–1.8926035**	**0.5796769**	**–1.1328303**	**0.7558361**	**3.6963476E–5**	**0.0065425355**
**hsa-miR-17**	**–2.2315624**	**0.6136431**	**–1.4382763**	**0.8383853**	**5.8513036E–5**	**0.0051784036**
hsa-miR-106b	–2.111961	0.9653531	–1.2075078	1.0892594	8.243697E–5	0.0048637814
hsa-miR-106a	–2.473755	0.826482	–1.5421348	0.89415276	1.7901328E–4	0.007921338
**hsa-miR-24**	**–2.1806908**	**0.66264117**	**–1.4844614**	**0.85892075**	**1.8698742E–4**	**0.0066193547**
hsa-miR-126	–1.6076399	0.47851408	–1.058029	0.5976432	2.0641266E–4	0.0060891733
**hsa-miR-16**	**–1.7805911**	**0.52026254**	**–0.99392307**	**0.8951935**	**2.244784E–4**	**0.005676097**
hsa-miR-222	–1.9393439	0.55772454	–1.3575824	0.74261785	2.3134724E–4	0.0051185577
hsa-miR-30c	–1.716693	0.8010705	–1.0352176	0.78270435	2.824945E–4	0.005555725
**hsa-miR-19b**	**–2.147926**	**0.90792555**	**–1.3004088**	**0.989784**	**2.913102E–4**	**0.0051561906**
hsa-miR-140-3p	–1.823895	0.56977737	–0.9603926	0.86705506	3.5182058E–4	0.005661113
hsa-miR-342-3p	–1.5989028	0.54853463	–1.0156938	0.5611862	4.4105874E–4	0.0065056165
**hsa-miR-20b**	**–2.1819031**	**0.76884466**	**–1.4245167**	**0.93230516**	**4.931529E–4**	**0.0067144665**
**hsa-miR-30b**	**–1.9062259**	**0.7578597**	**–1.2037946**	**0.75596815**	**5.4020213E–4**	**0.0068296986**
**hsa-miR-660**	**–2.2744212**	**0.7958325**	**–1.0648428**	**1.253039**	**5.7780603E–4**	**0.006818111**
**hsa-miR-25**	**–2.1231592**	**0.74814785**	**–1.2714314**	**1.2260581**	**6.002177E–4**	**0.0066399085**
**hsa-miR-20a**	**–2.4449308**	**1.0731456**	**–1.5648437**	**1.0846334**	**8.366445E–4**	**0.008710946**
hsa-miR-365	–0.6551892	0.55514914	–0.15434928	0.649751	8.380331E–4	0.008240659
**hsa-miR-331**	**–2.0739028**	**0.56269217**	**–1.3320043**	**0.84594274**	**9.056236E–4**	**0.008436599**
**hsa-miR-27a**	**–2.344583**	**0.88679594**	**–1.2909124**	**1.002102**	**0.0011549154**	**0.010221002**
hsa-miR-146b	–2.1507263	0.6010467	–1.5021411	0.75352633	0.0016511575	0.013916899
hsa-let-7d	–2.2001002	0.6704467	–1.534878	0.82270354	0.001878244	0.015111327
hsa-miR-195	–1.6405319	0.76942927	–0.9849392	1.1286806	0.0024855416	0.019127864
hsa-miR-142-3p	–1.6442593	0.698265	–1.0697229	0.9459838	0.0027333018	0.0201581
hsa-miR-146a	–2.0339553	0.52107155	–1.4923068	0.8067241	0.00367423	0.02601355
hsa-miR-150	–0.9646932	0.48798233	–0.5941478	0.6184291	0.0039196373	0.026683684
hsa-miR-199a-3p	–2.6115394	0.6630797	–1.9222051	0.98040193	0.0043441923	0.028478594
hsa-miR-574-3p	–2.2903006	0.7761696	–1.6703447	0.85751	0.004599253	0.029073851
hsa-miR-26a	–2.3018105	0.9024979	–1.8339038	1.1443821	0.005289506	0.032284226
hsa-miR-92a	–1.3904647	0.7350197	–0.8946887	0.78399193	0.0062127835	0.036655422
**hsa-miR-152**	**–2.1152809**	**0.77569246**	**–1.3102539**	**0.93023854**	**0.006841648**	**0.039063603**

(*Continued*)

microRNA	Mean expression at Dx	SD at Dx	Mean expression at Remision	SD at Remision	Adjusted *p*-value	Fold change
hsa-miR-301	–2.384497	0.43514875	–1.639337	0.8616541	0.006903818	0.038186744
**hsa-miR-18a**	**–3.0904574**	**0.4145374**	**–2.2442312**	**1.0040047**	**0.0074316533**	**0.03986069**
hsa-miR-361	–2.345653	0.32122228	–1.781628	0.7330183	0.0075109918	0.03910134
hsa-miR-223	–2.2401733	0.61627126	–1.5986383	1.0725389	0.007991364	0.040413473
hsa-miR-486	–1.1674733	0.6841681	–0.57494414	0.83149815	0.008036599	0.03951328
hsa-miR-19a	–2.0261908	0.5908374	–1.3119819	1.0760777	0.008133381	0.038908333
hsa-miR-130a	–2.7822578	0.52694046	–1.8854575	1.100752	0.008425139	0.039243408
hsa-miR-484	–1.4542063	0.38707164	–1.0252968	0.6013224	0.008460162	0.03839612
hsa-miR-532-3p	–2.1023054	0.6293188	–1.3739631	0.94726264	0.009743341	0.043114286
